# Luteal phase support in intrauterine insemination cycles

**DOI:** 10.4274/tjod.89577

**Published:** 2016-06-15

**Authors:** İsmet Gün, Özkan Özdamar, Ali Yılmaz

**Affiliations:** 1 Gülhane Military Medical Academy, Haydarpaşa Training and Research Hospital, Clinic of Obstetrics and Gynecology, İstanbul, Turkey; 2 İstanbul Medeniyet University Faculty of Medicine, Department of Obstetrics and Gynecology, İstanbul, Turkey

**Keywords:** intrauterine insemination, controlled ovarian stimulation, luteal phase support

## Abstract

Intrauterine insemination (IUI) treatment aims to increase the rate of conception by increasing the chances that the maximum number of healthy sperm reach the site of fertilization. IUI with controlled ovarian stimulation is frequently used in assisted reproduction practice. Although widely used, the efficacy of luteal support in IUI remains controversial. In this article, we aimed to review what we know regarding luteal support in IUI cycles and to adjudicate about the clinical use and benefits of this treatment. Based on the study results available in the literature, it appears to be beneficial to supplement the luteal phase in gonadotropin-stimulated IUI cycles that yield more than one follicle.

## PRECIS:

Based on the study results in the literature, it appears to be beneficial to supplement the luteal phase in gonadotropin-stimulated intrauterine insemination cycles that yield more than one follicle.

## INTRODUCTION

## THE AIM, FREQUENCY, AND SUCCESS RATE OF INTRAUTERINE INSEMINATION

Intrauterine insemination (IUI) is an artificial insemination technique in which sperm is introduced directly into the uterine cavity irrespective of whether ovulation has been triggered. The purpose of this process is to increase the rate of conception by improving the sperm quality and concentration and by transporting the maximum number of healthy sperm to the site of fertilization. Controlled ovarian stimulation (COS), which involves a variety of ovulation induction (OI) agents, is used before the procedure in an effort to increase the number of oocytes, eliminate ovulation disorders that are not detected during regular examinations, and provide the optimal conditions for insemination.

Although the cost-effectiveness of IUI has been questioned, it is a widely-used fertility treatment that gives patients a good chance of pregnancy with relatively lower cost as compared with in vitro fertilization/intracytoplasmic sperm injection (IVF/ICSI); however, the difference between the two is significant. Within the context of the European IVF Monitoring Programme in 2004, data from 19 countries were analyzed. It was reported that IUI was performed in 98.388 of a total of 245.099 cycles, or 40.1% of infertile patients, (52.866 IVF cycles and 93.845 ICSI cycles), and 12.081 births (12% per cycle) were reported in these IUI cycles, with a multiple pregnancy rate of 13%. The same data indicated that the number of donor inseminations was relatively high (with a 2004 donor insemination cycle number of 17.592, a pregnancy rate per cycle of 17.7%, and a multiple pregnancy rate of 11.8%)^(1)^. The European Society of Human Reproduction and Embryology (ESHRE) also reported that IUI practices in Europe have increased over the years, and 162.843 IUI cycles with 29.235 donor cycles for IUI were carried out in 2009^(2)^. This outcome is not surprising given that IUI is employed in a broad range of indications including mild male infertility, unexplained infertility, and minimal or mild endometriosis. The effectiveness of IUI depends on a set of variables including the extent of IUI use, the indications for IUI, the optimal procedures for sperm preparation, insemination methods and timing, and the need to prevent premature luteinizing hormone (LH) surges and luteal deficiency in stimulated IUI cycles^(3)^.

The main problem with IUI cycles with respect to the data in question is that pregnancy rates per cycle are lower as compared with IVF/ICSI, for which there are many possible reason. The leading one may be that the amount of research focused on IUI is not sufficient. A literature search on PubMed using the phrase IUI revealed that the first publication on this issue was published by in 1962 and also yielded 2.233 publications since October 2015. In contrast, when PubMed is searched using the phrase IVF, the resulting number of hits is 38.340. There are nearly 17 times as many IVF studies as there are IUI studies. Consequently, these data indicate that although frequently employed, IUI is not adequately studied. For this reason, we suppose that there are many unknowns to be analyzed. One of which is whether luteal phase support (LPS) is necessary for IUI cycles. When the current literature was investigated using the phrase LPS in IUI, PubMed yielded 42 publications since October 2015 on this subject. These publications include two meta-analyses in 2013 and very few controlled prospective studies to date. Moreover, the Cochrane database does not have any articles on the topic.

## KNOWN FACTS AND OBSERVATIONAL STUDY RESULTS

An LH surge is needed for follicle rupture and oocyte maturation. Similarly, progesterone (P) is needed to support early pregnancy and implantation. P is a product of the corpus luteum (CL) and provides secretory transformation of the endometrium during the luteal phase. In order for P receptors to diffuse sufficiently, a sufficient amount of estrogen (E) is needed^(4)^. In all COS cycles, multiple follicular development and the resultant supraphysiologic estradiol block the hypothalamic-pituitary axis with negative feedback, and hence the necessary LH oscillation for CL function does not occur. Consequently, this situation causes defective P and premature luteolysis^(5)^.

Analysis of the available observational studies revealed that the luteal phase in gonadotropin-stimulated cycles is 20% shorter (an average luteal phase lasts 11 days), and that this shortness can be normalized by administering mid-luteal human chorionic gonadotropin (hCG), and that groups receiving LPS had significantly higher levels of mid-luteal P than those receiving no support^(6,7,8,9)^. However, mid-luteal P levels were <10 ng/mL in only one-third of the cycles that had a shortened luteal phase. This demonstrates that mid-luteal P levels are not directly related with luteal function^(6)^. Additionally, P plays a crucial role in LH secretion modulation, and long-term exposure to P or E+P can reduce LH secretion^(10,11)^.

Exogen hCG administration can reduce LH concentrations through short and long feedback mechanisms^(12)^. However, more comprehensive studies demonstrated that hCG injection does not induce down-regulation of LH secretion during the luteal phase in normoovulatory women in spite of multifollicular development in unstimulated cycles and therefore supraphysiologic steroid concentration^(13)^. On the other hand, ovarian stimulation and the related multifollicular development are associated with abnormal endometrium progression during the early luteal period in almost 50% of cases^(14)^.

## PREMATURE LUTEINIZING HORMONE SURGE IN INTRAUTERINE INSEMINATION CYCLES

An LH surge is triggered by the increasing levels of E secreted by the dominant follicle and is a requirement for follicular rupture and oocyte maturation. A premature LH surge is defined as a premature rise of LH (>10 IU/L) accompanied by a concomitant rise in P (>1 mg/L-3.2 nM/L)(15). With the exception of natural cycles in older women, premature LH surge is a rarely-encountered phenomenon, although in stimulated IUI cycles its rate approaches 25-30%^(15,16)^. Premature LH surges may result in cycle cancellation or treatment failures. Gonadotropin-releasing hormone antagonists correct the premature LH surge but do not affect pregnancy rates, which indicates that it is not completely a case of early luteinization or premature LH surge^(15)^.

## META-ANALYSIS AND PROSPECTIVE RANDOMIZED CONTROLLED STUDIES FOR LUTEAL PHASE SUPPORT IN INTRAUTERINE INSEMINATION CYCLES

Despite some controversies in the published literature, luteal deficit and its causes are clarified thanks to progress in assisted reproduction technique. From this perspective, the usefulness of P supplementation during the luteal phase in IVF/ICSI cycles for reproductive outcomes is notably accepted in the Cochrane study. However, the timing of initiation, duration, route and amount of administration are still debated^(17)^. On the other hand, the effects of LPS in IUI cycles are unclear. To date, a very limited number of randomized controlled prospective studies has been conducted regarding the necessity of LPS treatment in stimulated IUI cycles. IUI cycles resemble IVF cycles in terms of multifollicular production and represent supraphysiologic steroid production. In this regard, it could be plausible to consider that the number of follicles in stimulated IUI cycles does matter and that LPS may be needed in multifollicular cases.

The relevant literature investigation with regard to stimulated IUI cycles + LPS produced only two studies that addressed the issue of follicle number. The first is the report of ESHRE Capri Workshop Group in 2009, which assessed six randomized controlled studies including 456 patients^(3)^. In this report, it was stressed that LPS treatment was not a major requisite for mild gonadotropin-stimulation IUI cycles (1-2 follicles). The second was the study by Seckin et al.^(18)^ in 2014, which compared women who received a vaginal P gel with controls in an unexplained infertility population undergoing gonadotropin-stimulated IUI cycles. According to the results of this study, it was suggested that there were no difference between groups that received and did not receive LPS in terms of clinical pregnancy rates (CPR) and live birth rates (LBR) per cycle and per patient; however, in the IUI cycles that yielded a multifollicular response (>1 dominant follicle), LPS treatment statistically increased the CPR per patient compared with those with monofollicular response (28.2% vs. 11.4%, respectively, p=0.04). This result was the first evidence that LPS affected the success of the multifollicular resulst in gonadotropin-stimulated IUI cycles.

A literature search with regards to prospective randomized controlled trials (RCT) revealed the first study was conducted by Erdem et al.^(19)^ in 2009. In this study, groups receiving and not receiving vaginal P gel for LPS two days after IUI in rFSH-stimulated IUI cycles in patients with unexplained infertility were compared. The results revealed that both CPR per cycle (21.1% and 12.7%, p=0.028, respectively) and per patient (39.4% and 23.8%, p=0.01, respectively) and LBR per cycle (17.4% and 9.3%, p=0.016, respectively), and per patient (35.8% and 18.1%, p=0.003, respectively) were significantly higher in the LPS-receiving group. Multiple pregnancy rates (MPR) did not significantly differ between the groups. This was the first prospective randomized controlled study to be conducted in this field that provided live birth rates. To date, there have been two meta-analyses,^(20,21)^ including five RCTs^(19,22,23,24,25)^ regarding this issue. Although all five RCTs included in the two meta-analyses provided biochemical pregnancy rate (BPR), CPR, MPR, and miscarriage rates (MR), the studies conducted by Erdem et al.^(19)^, Ebrahimi et al.^(23)^, and Maher^(24)^, also assessed LBR. None of the studies indicated a difference between MPR and MR. Vaginal P was used for LPS in all these studies. Meta-analyses by Hill et al.^(20)^ and Miralpeix et al.^(21)^ revealed significantly higher CPRs [OR: 1.47; 95% CI: (1.15-1.98) and RR: 1.41; 95% CI: (1.14-1.76), respectively] and LBRs [OR: 2.11; 95% CI: (1.21-3.67) and RR: 1.94; 95% CI: (1.36-2.77), respectively] in LPS-administered groups as compared with LPS-free groups. In the subgroup analyses, LPS was reported to significantly increase BPR, CPR, and LBR only in the gonadotropin-stimulated IUI cycles compared with the LPS-free group. However, none of the studies present data regarding the number of follicles yielded. In addition, Miralpeix et al.^(21)^ categorized the five studies assessed in their meta-analyses as either low risk of bias (if all the questions were answered yes) or high risk of bias (if at least one question was answered no) with respect to the responses to six parameters, which were “sequence generation, allocation concealment, blinding, incomplete outcome data, selective outcome reporting and other issues.” Consequently, studies conducted by Kyrou et al.^(22)^ and Ebrahimi et al.^(23)^ were defined as having a high risk of bias, whereas the others had a low risk of bias.

After the above-mentioned meta-analyses, two more double-blinded prospective randomized controlled studies were published, both of which were conducted in Iran. The first was a placebo-controlled study in 2014 by Hossein Rashidi et al.^(26)^ which involved vaginal P for LPS administered until the 8^th^ gestational week. COS was achieved with clomiphene citrate (100 mg/d) and human menopausal gonadotropin (75 IU/d) in the study groups. No statistically significant difference was detected between the groups receiving LPS (n=127) and placebo (n=126) in terms of BPR (30.8-22.2%, p=0.15, respectively), CPR (15.8-12.7%, p=0.30, respectively), MR (10-18.8%, p=0.45, respectively) and the ongoing pregnancy rate (OPR) (46.2-46.4%, p=0.98, respectively). The second study was conducted in 2015 by Khosravi et al.^(27)^ in which vaginal P (400 mg) and oral dydrogesterone (20 mg) groups were compared for LPS with regards to CPR, MR, and mid-luteal P values (seven days after IUI). Although there was no statistical difference, the mid-luteal P level in the oral dydrogesterone arm was higher and, accordingly, MR was lower. Consequently, this study demonstrated that oral dydrogesterone could also be effective for LPS as vaginal P in COS+IUI cycles. The characteristics and reproductive outcomes of the RCTs available in the literature are demonstrated in detail in Tables 1 and 2.

## CONCLUSION

In conclusion, COS+IUI cycles are similar to IVF/ICSI cycles in terms of multifollicular development. Only a limited number of randomized controlled trials are available about LPS in IUI cycles. Based on the results of these studies, it appears to be beneficial to support the luteal phase in gonadotropin-stimulated IUI cycles that yield more than one follicle. There is still a need for further randomized controlled trials to evaluate the effectiveness of LPS treatment in stimulated IUI cycles.

## Figures and Tables

**Table 1:**
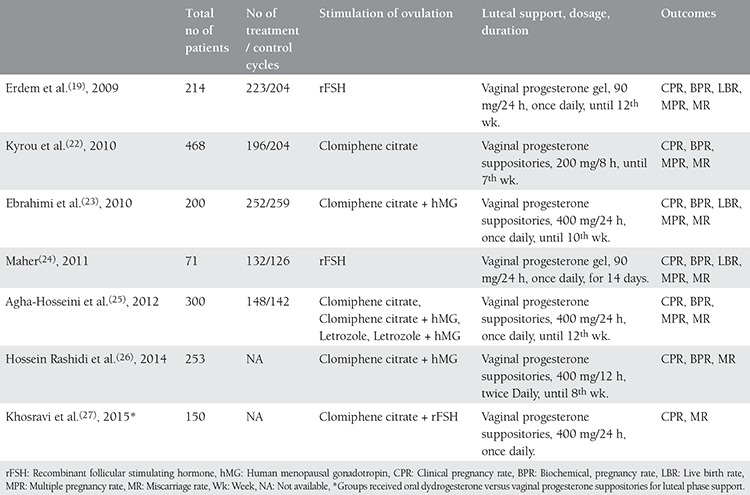
Characteristics of the randomized controlled trials that assessed luteal phase support in women undergoing intrauterine insemination cycles

**Table 2:**
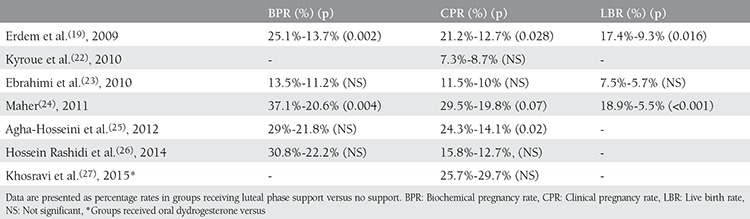
Reproductive outcomes of randomized controlled trials that assessed luteal phase support in women undergoing intrauterine insemination cycles
